# Using isoelectric point to determine the pH for initial protein crystallization trials

**DOI:** 10.1093/bioinformatics/btv011

**Published:** 2015-01-07

**Authors:** Jobie Kirkwood, David Hargreaves, Simon O’Keefe, Julie Wilson

**Affiliations:** ^1^Department of Chemistry, University of York, YO10 5DD, UK, ^2^Discovery Sciences, Structure & Biophysics, AstraZeneca Darwin Building, Cambridge, Science Park, Milton Road, Cambridge, CB4 0WG, UK, ^3^Department of Computer Science, University of York, YO10 5GH, UK and ^4^Department of Mathematics, University of York, YO10 5DD, UK

## Abstract

**Motivation:** The identification of suitable conditions for crystallization is a rate-limiting step in protein structure determination. The pH of an experiment is an important parameter and has the potential to be used in data-mining studies to help reduce the number of crystallization trials required. However, the pH is usually recorded as that of the buffer solution, which can be highly inaccurate.

**Results:** Here, we show that a better estimate of the true pH can be predicted by considering not only the buffer pH but also any other chemicals in the crystallization solution. We use these more accurate pH values to investigate the disputed relationship between the pI of a protein and the pH at which it crystallizes.

**Availability and implementation:** Data used to generate models are available as Supplementary Material.

**Contact:** julie.wilson@york.ac.uk

**Supplementary information:** Supplementary data are available at *Bioinformatics* online.

## 1 Introduction

As the predominant method used to determine molecular structure, X-ray crystallography can provide information on the atomic level processes of proteins and, in turn, enable the development of therapeutic drugs. In order to determine the structure of a target protein by X-ray crystallography, the protein must be cloned and expressed before the soluble fraction is purified for crystallization. Only when diffraction quality crystals are obtained can the structure be determined. Although many protein structures have been solved to date, these only account for some 3% of all possible protein targets (TargetDB, 2010), with membrane protein structures being particularly challenging (Caffrey, 2003) and underrepresented (Doerr, 2008). The use of a priori information about a protein to ascertain the optimal initial conditions before experimentation could help reduce the number of trials required which is valuable when protein sample availability is scarce. Following the advent of online repositories, such as the Protein Data Bank (PDB) (Berman *et al.*, 2000) and the Biological Macrmolecule Crystallization Database (Tung and Gallagher, 2008) set up specifically for the development of crystallization strategies (Gilliland *et al.*, 1994), it has been possible to analyse the data from successful experiments and determine favourable regions of crystallization parameter space.

It has been shown that protein family can indicate a region of parameter space in which a protein is likely to crystallize (Hennessy *et al.*, 2000) and that properties such as amino acid frequency, sequence length, grand average of hydrophobicity (GRAVY) (Kyte and Doolittle, 1982) and isoelectric point (pI) as well as other properties can determine a protein’s propensity to crystallize (Smialowski *et al.*, 2006; Chen *et al.*, 2007; Overton *et al.*, 2008). Investigations have also indicated that the isoelectric point could be used to determine the pH at which a protein with an acidic isoelectric point is likely to crystallize (Kantardjieff and Rupp, 2004; Charles *et al.*, 2006).

Crystallization is often described as a two-stage process, with the first step being identification of the conditions that allow crystallization, followed by the optimization of these conditions to obtain diffraction-quality crystals (Luft *et al.*, 2003; McPherson, 2004). This study focuses on the first stage—the identification of conditions that encourage crystallization. An analysis of 9596 structures obtained from the PDB suggested a link between a protein’s pI and the pH at which it would crystallize. It was found that acidic proteins tended to crystallize 0–2.5 pH units above their pI, whereas basic proteins crystallized 0.5–3 pH units below their pI (Kantardjieff and Rupp, 2004). The authors reported a correlation between pI and pH–pI that was challenged with claims that the predictive statements had been made using a misinterpretation of the data (Huber and Kobe, 2004). As a form of data normalization, there will always be a link between pI and pH–pI, but it was also highlighted that no correlation between pI and pH had been found previously (Page *et al.*, 2003; Wooh *et al.*, 2003). In defence of their work, the authors of the original study showed a correlation between the pI of acidic proteins and the pH of successful crystallization and that a linear model could be used to predict the optimal pH for such proteins. However, they concluded that a similar model could not be created for basic proteins because no significant correlation was found (Kantardjieff *et al.*, 2004). Since the original study, similar relationships between the pI of proteins and the buffer pH of successful crystallization experiments have been noted for both acidic and basic proteins (Charles *et al.*, 2006).

The isoelectric point determines a protein’s minimum solubility level due to protein–protein interactions being favoured over protein–water interactions (Gilliland, 1988; Luft *et al.*, 2011). It should therefore follow that a solution with a pH matching the isoelectric point would be ideal for crystallization although this has never been confirmed. One possible reason for this is that the recorded pH is that of the buffer in the crystallization solution rather than the final pH of the crystallization cocktail (Zhang *et al.* 2013). It is known that the reported buffer pH can differ from the true pH of the experiment by more than three pH units (Bukrinsky and Poulsen, 2001; Newman *et al.*, 2012).

Measurement of pH using a pH meter is time consuming and can be impractical if it requires reformatting of the crystallization screen to accommodate the probe. We have shown previously that accurate measurements of pH can be obtained for crystallization solutions using spectrophotometry (Kirkwood *et al.*, 2014). The values determined by our spectrophotometric method are typically within 0.2 pH units of those measured using a pH meter. Here, we use the spectrophotometric pH values obtained from numerous experiments to train a neural network to assign pH values to crystallization conditions. These values are shown to provide accurate estimates of the pH that can be used, for example, when mining databases such as the PDB. Using data obtained from AstraZeneca and from the Structural Genomics Consortium (SGC), Oxford, we show that most proteins, both acidic and basic, do crystallize within one unit of their isoelectric point. This in turn allows for custom crystallization screens to be developed in instances where protein availability is scarce and allows deeper exploration of chemical parameter space as the pH is fixed.

## 2 Materials and methods

### 2.1 The AZ dataset

Table 1 gives details of the 14 proteins, some commercially available and some in-house targets from AstraZeneca, which were screened over a range of pH values. Commercial proteins were obtained from Sigma-Aldrich and were buffered at pH 7.6. In-house proteins were also buffered at near neutral pH (either pH 6.5 or pH 7.5). In order to determine the conditions for crystallization, each protein was initially screened using sitting-drop vapour diffusion with a bespoke 96 condition sparse matrix screen buffered at 6 different pHs using the multi-component buffer PCTP (Newman, 2004; Zhang *et al.*, 2013). This gave a total of 576 conditions with the buffer pH fixed between pH 4.5 and pH 9.5. For each well, 80 μl of crystallization solution was dispensed using a Thermo Scientific Matrix Hydra II robot. Frozen protein samples were defrosted to room temperature before using a Mosquito pipetting robot (TTP Labtech) to dispense 1 μl protein with 1 μl of the mother liquor in MRC Wilden crystallization trays. Trays were sealed manually using transparent, pressure-sensitive adhesive tape (Hampton) and stored in a Formulatrix Rock Imager hotel at 20°C. All images were assessed for crystallization after 21 days and the best crystallization conditions selected for a particular protein. A finer sampling of pH was performed in a 96-well plate with the chosen components buffered between pH 4.5 and pH 9.5 with PCTP.

**Table 1.** AstraZeneca dataset details

(a)				
Protein	Source*	Conc. (mg/ml)	Buffer solution	pI
Protease K212A	a	13	1	4.93
Protease K234A	a	13.4	1	5.03
Protease K249A	a	12.1	1	5.03
ProteaseE171A	a	13.2	1	5
Concanavalin A	b	15	2	5.47
Bovine catalase	b	15	2	6.79
Pig Trypsin	b	31	2	7
Thaumatin	b	50	2	8.46
α- Chymo A	b	15	2	8.52
Lysozyme	b	20	2	9.36
Glycolytic A	a	30.1	3	7.52
Glycolytic D	a	21.9	3	6.75
Glycolytic wt	a	9.76	3	6.75
Kinase 1	a	12.2	4	5.18

(b)				

1	20 mM MES, 5 mM calcium chloride, 5 mM DTT, 100 mM sodium chloride, 300 mM AHA, pH 6.5			
2	10 mM PCTP, 100 mM sodium chloride, 0.5 mM TCEP, pH 7.6			
3	20 mM Tris–HCL, 150 sodium chloride, 2 mM TCEP, pH 7.5			
4	10 mM Tris, 50 mM sodium chloride, 1 mM DTT, 50 µM Zinc Acetate, pH 7.5			

*Note*: pI values for the commercially available and in-house protein targets that were screened over a range of pH values are shown in (a) with buffer solution details in (b). Source: a, In-house; b, Sigma-Aldrich.

### 2.2 The SGC dataset

The data obtained from the SGC in Oxford relate to 62 605 constructs, from which 608 solved protein structures have been deposited in the PDB. Descriptive metadata include protein sequence, family and purification methods as well as crystallization conditions and follow-up data with PDB codes where relevant. A subset of data were extracted for experiments performed using the same SGC − JCSG +4 sparse matrix screen. The extracted data comprised the protein sequence and experimental conditions for 1057 different protein sequences. Experimental results were assessed using the score given by a crystallographer, together with the resolution of the diffraction data and whether or not the structure was solved. For crystals that were not of diffraction-quality, no estimated resolution is given it was assumed that the structure was not determined. In instances where crystals were found to be salt, the associated data were removed. Data from unbuffered experiments were also removed as it is not possible to obtain an accurate pH value for such solutions. The remaining data were grouped based on the final stage reached in the structure determination pipeline as follows:

Group 1: 61 sequences that resulted in structure determination.
Group 2: 50 sequences that resulted in a crystal that diffracted to at least 3.6 Å.
Group 3: 211 sequences that resulted in at least one protein crystal.
Group 4: 735 sequences that were annotated as ‘crystal—to be followed up’.

It should be noted that sequences in the final group may not relate to diffraction quality crystals or could be salt crystals that had not yet been identified as such. Conversely, it is possible that perfectly good crystals may have been overlooked.

### 2.3 Calculation of isoelectric point

Proteins can become more positively or negatively charged by gaining or losing protons due to the pH of their environment. The isoelectric point (pI) is the pH at which a protein has a net charge of zero and can be calculated using the charges for the specific amino acids in the protein sequence. Estimated values for the charges are called acid dissociation constants or *pK_a_* values. In the following analysis, the *pK_a_* values used are those used in the EMBOSS software suite (Rice *et al.*, 2000) as shown in Table 2. For a protein with *n*− negatively charged amino acids and *n*+ positively charged amino acids, the pI can be determined as the pH for which the net charge given by Equation (1) is zero:

(1) $$\text{net charge}=\overset{n_{-}}{\underset{i=1}{\sum }}\frac{-1}{1+10^{pK_{n}-pH}}+\overset{n_{+}}{\underset{i=1}{\sum }}\frac{1}{1+10^{pH-pK_{p}}},$$

where *pK_n_* and *pK_p_* are the *pK* values for negatively charged and positively charged amino acids, respectively. As an example, consider the small amino acid sequence, CRV, with one cysteine (*pK_n_* = 8.5), one arginine (*pK_p_* = 12.5) and one valine (no charge). Including the N-terminal amine group (*pK_p_* = 8.6) and C-terminal carboxyl group (*pK_n_* = 3.6) the net charge for an initial pH of 0 is given by Equation (2):

(2) net charge= −11+103.6−0+−11+108.5−0+11+100−12.5+11+100−8.6≈0+0+1+1≈2.

**Table 2.** EMBOSS acid dissociation constants

Amino acid	*pK_a_*	Charge
Amine group	8.6	Positive
Carboxyl group	3.6	Negative
Cysteine (C)	8.5	Negative
Aspartic acid (D)	3.9	Negative
Glutamic acid (E)	4.1	Negative
Histidine (H)	6.5	Positive
Lysine (K)	10.8	Positive
Arginine (R)	12.5	Positive
Tyrosine (Y)	10.1	Negative

**Fig. 1.**
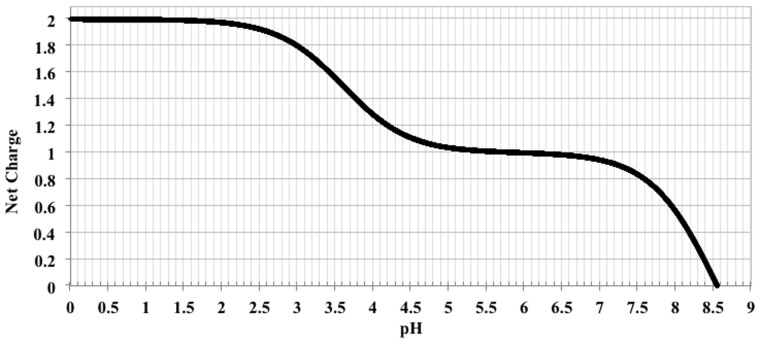
Net charge of the sequence CRV with varying pH

Therefore, the charge for the sequence CRV is approximately 2 at pH 0. By gradually increasing the theoretical pH, to make the net charge in Equation (1) equal to zero, it is found that the isoelectric point of CRV is 8.555, as shown in Figure 1.

The isoelectric point for each sequence in the SGC dataset was determined in the same manner using an Excel spreadsheet with visual basic for applications (Microsoft VBA). For the AZ dataset, the pI was either obtained from Zhang *et al.* (2013) or calculated as above and was confirmed using isoelectric focusing.

### 2.4 Determination of pH by spectrophotometry

A method to accurately determine pH using spectrophotometry has been developed and is described in detail elsewhere (Kirkwood *et al.*, 2014). Briefly, the method uses spectrophotometry to measure light absorbance when the indicator dye, bromothymol blue, is mixed with the crystallization solution. The absorbance is measured between 400 and 700 nm in 5-nm increments and the curve obtained compared with a set of curves for conditions of known pH. The pH of the most similar curve, determined by the smallest mean absolute deviation, is assigned as the pH of the condition in question.

Spectrophotometry was used to determine pH values for buffered conditions in a variety of crystallization screens, including the JCSG-Plus, the Rigaku Wizard, the Hampton Index and the JCSG + 6, an evolution of the JCSG +4 screen—the one used in the analysis of the SGC data. However, as the spectrophotometry method is limited to values in the range from pH 4.5 to pH 9.5, all conditions that were assigned a pH value of either 4.5 or 9.5 were removed from the dataset to avoid the possibility of under or overestimating the true pH at the extremes. For each of the remaining 5161 conditions, the chemicals and their concentrations together with the pH measured by spectrophotometry are given in Supplementary Data S1.

### 2.5 Linear regression modelling of pH

**Fig. 2.**
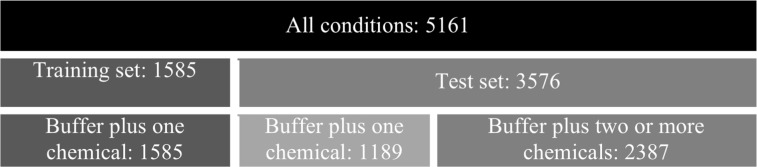
Organization of data used for linear regression modelling

In the absence of measurements obtained through a pH meter or spectrophotometry, pH becomes a problematic parameter in data mining as its inaccuracy could lead to misleading conclusions. However, it is possible that an accurate estimate of pH can be obtained without experimentation through the use of regression modelling. The dataset comprising 5161 spectrophotometric pH values together with the buffer pH and the concentrations of the chemical species involved was divided into a training set, consisting of those conditions with only one chemical species in addition to the buffer, and a test set of the conditions with multiple chemical species (Fig. 2). It was found that a linear regression model of the form:

(3) $$\hat{pH_{S}}=\beta_{0}+\beta_{1}B+\beta_{2}{\text{log}}_{10}C+\beta_{3}Bċ{\text{log}}_{10}C,$$

where $\hat{pH_{S}}$ is the predicted pH, *B* is the buffer pH, *C* is the concentration and the *β* terms are the regression coefficients, was suitable for each chemical species. Inspection of the regression coefficients for individual chemical species revealed patterns in the models, with subsets of chemicals having similar regression coefficients and the same predictor variables shown to be insignificant. Supplementary Table S1 shows how groups with similar regression models also share similar chemistry. Final regression models were calculated for each group, after removing 10% of the data from each group for validation. This grouping of chemicals not only provides a more reliable predictive model due to the increased sample size, but it also allows new chemicals, which are not present in the training set, to be assigned to a group and an estimate of pH obtained.

For solutions containing multiple chemical species, pH values were obtained by combining the predicted pH values for each individual chemical at the appropriate concentration using the formula:

(4) $$\hat{pH_{A}}=-{\text{log}}_{10}\left(\frac{\sum_{i=1}^{n}10^{-{\hat{pH}}_{s_{i}}}}{n}\right),$$

where $\hat{pH_{A}}$ is the predicted pH for the solution containing all elements, *n* is the number of chemical species in the solution and $\overset{}{pH_{S_{i}}}$ is the predicted pH the individual species, *S_i_*. The formula effectively determines the pH value by averaging the number of hydrogen atoms for each chemical in the solution. The 10-fold increase in hydrogen ions per pH unit decrease shows that the pH of the solution is dominated by the most acidic species, which is modified slightly by more basic species. The model requires no weighting of the parameters as the concentration of individual chemicals has already been accounted for.

### 2.6 Modelling pH using machine learning

An artificial neural network (ANN), implemented in Matlab (MathWorks, 2011) was trained to assign a pH value to crystallization solutions. An ANN was employed as they are able quickly to create a richer, non-linear model than that of regression. ANNs are machine learning algorithms designed to mimic the processes in the brain and can be trained to associate a particular output, in this case a pH value, with particular input features. The neural network was implemented in Matlab using the fitnet function with the Levenberg–Marquardt back-propagation method and the hyperbolic tangent sigmoid transfer function (Beale and Jackson, 1990; MathWorks, 2013). A single hidden layer network was chosen to reduce the risk of overfitting as regression modelling had shown that a linear model was sufficient. Approximately two-thirds of the data for the 5161 conditions for which pH values could be determined by spectrophotometry were used to train the network and the other third was reserved as an independent test set. The chemical species included salts, salts of weak acids, organics, polyethylene glycols (PEGs) of different molecular weights and different functional groups, compounds containing ammonia, hydroxide and di-hydrogen salts. Chemicals were broadly grouped as suggested by the linear regression analysis (Supplementary Table S1) and stratified sampling used to divide the chemical groups evenly between the training and test sets (3524:1637). The concentration of chemicals in each group was calculated for each condition and these values, together with the buffer pH, used as inputs to the neural network. We chose a network with a single hidden layer of five nodes as this was the simplest network that gave a low mean squared error (MSE) between the output pH and the spectrophotometric pH during training without overfitting (as assessed by the independent test set).

## 3 Results

### 3.1 Assignment of pH

Linear regression showed that four chemical groups—ammonia, acids, basics and salts of acids—require the full model including the interaction term relating both the buffer pH and the additional chemical concentration to the pH of the experiment. The model for PEGs does not include the chemical concentration as a separate term, but does include the interaction between chemical concentration and buffer pH. Organics and salts have the simplest models, only involving the buffer pH as a variable. The simplified models were obtained by stepwise variable selection using Bayesian Information Criterion. More details are given in the Supplementary Material. The MSE between the spectrophotometric and predicted pH values is 0.28 in comparison to 0.8 between the values measured by spectrophotometry and the buffer pH values. The (Pearson’s product moment) correlation with the measured values is 0.89 for the predicted pH in comparison to 0.77 for the buffer pH.

**Fig. 3.**
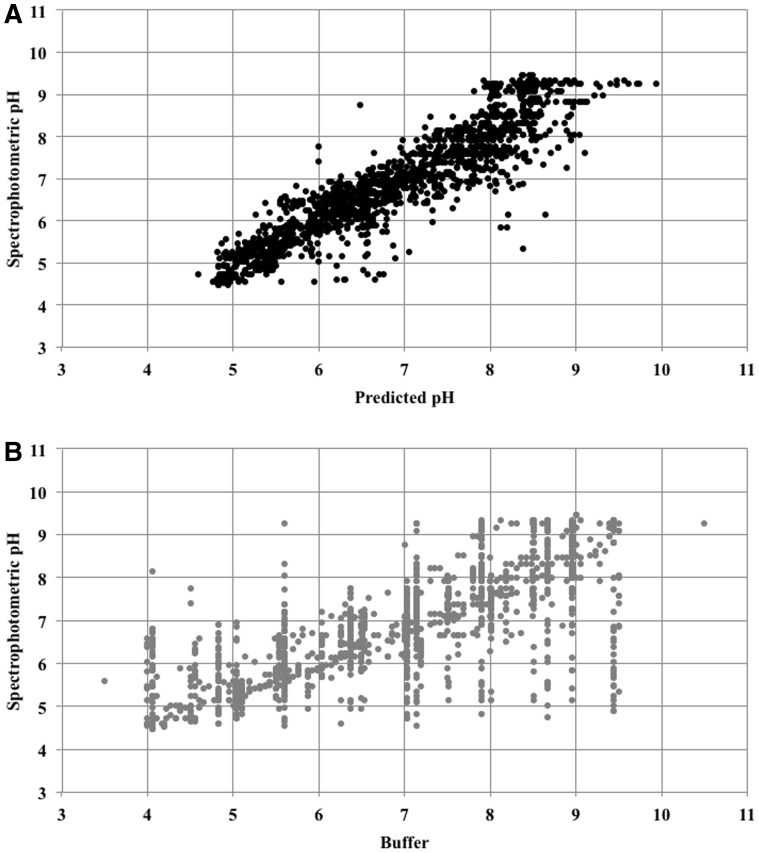
Accuracy of pH values. The pH obtained by spectrophotometry is shown plotted against the pH predicted by an artificial neural network in (A) and the pH of the buffer in (B)

Figure 3a shows the pH values measured by spectrophotometry plotted against those predicted by the neural network for the independent test set. The linear relationship between measured and predicted pH can be shown to have an intercept close to 0 and a gradient close to 1 suggesting a strong relationship between the two methods of obtaining pH. For the same test data, the spread of values obtained by spectrophotometry for any particular buffer pH is much greater than for the corresponding predicted pH, as can be seen in Figure 3b. The correlation of the spectrophotometric pH with the predicted pH is 0.92 (MSE 0.25) in comparison to 0.75 with the buffer pH (MSE 0.97).

**Fig. 4.**
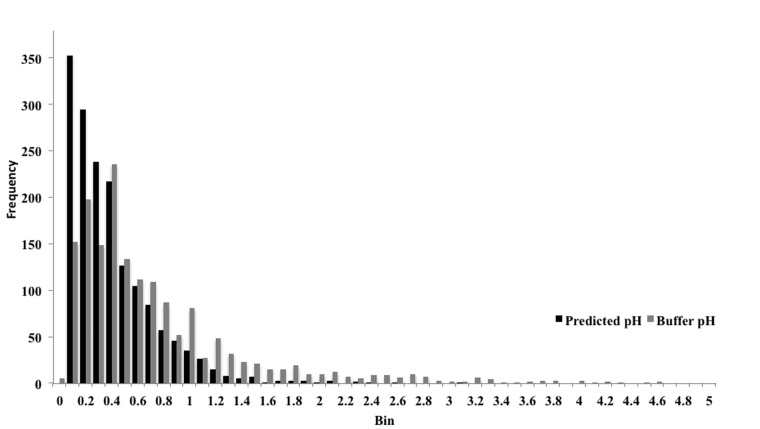
Histogram showing errors in predicted and buffer pH values in relation to the spectrophotometric pH

The distribution of differences from the spectrophotometry pH values is shown in Figure 4 for the values assigned by the neural network and for those provided by the buffer pH. The histograms show the absolute deviations in 0.1 pH unit bins. Although 75% of predicted pH values are within 0.5 units of the measured pH (i.e. ±0.5 pH units) and 95% are within one unit, only 53% of the buffer pH values are within 0.5 units and just 80% are within one unit.

Closer inspection of the predicted values reveals that 6 of the 66 individual chemicals were involved in the conditions where the deviation from the spectrophotometric pH values was unusually high. One of these chemicals, PEG 2000 DME, should be neutral, but spectrophotometry suggested a pH of just over 4.5, at the limit of the method’s reliability. However, it is known that PEGs degrade, becoming more acidic over time (Ray and Puvathingal, 1985; Jurnak, 1986; Hampton, 2012). Indeed, when checked with a Jenway 4330 pH meter, the solution was found to have a pH of just 2.6. The other five chemicals that were associated with large errors (jeffamine ed-2003, ammonium phosphate dibasic, dl-malic acid, sodium malonate, magnesium chloride) were not well-represented in the training data. Retraining the network with a larger dataset could therefore improve the results further.

Throughout we have used models to predict the pH of the crystallization solution, however, only a proportion of this is contained within the drop containing the protein. Using a lysozyme solution at 40 mg/ml with 100 mM sodium chloride and the buffer PCTP at 50 mM, we have shown that, when mixed with 50 mM PCTP at pH 5, 7 and 9, the final pH could be predicted from the two buffering components with neither the salt or the lysozyme having a noticeable effect. For example, protein solution at pH 5 to mother liquor pH 7 in the ratio 2:1 gives a predicted pH of 5.66 which compares with an average measured pH of 5.46 (as shown in Supplementary Table S3). Only when the ratio of protein solution:mother liquor was increased to 3:1 did we find that the lysozyme affected the pH.

### 3.2 Relationship between pI and pH

In order to investigate the claims regarding a relationship between the pI of a protein and the pH at which it can be crystallized, we calculated the pI from each protein sequence in the SGC dataset. These data, all screened using the SGC − JCSG + 4 sparse matrix screen, were selected from the full SGC database and assigned to chemical groups in order to predict pH as shown in Supplementary Table S2. Although a spectrophotometric pH value was available for some of the conditions in the SGC − JCSG + 4 screen (used either for training the neural network or reserved to test the accuracy of the assignments), the pH used here for all conditions was that assigned using the trained neural network. In addition to the chemical concentrations, the pH of the crystallization buffer is used as input to the network. For those wells without a buffer solution (21/96), the pH of the purification buffer was used instead. Data for any wells where neither buffer pH nor purification pH was available were removed. It has been shown that the buffering capacity of the protein itself is negligible both in vitro (Kirkwood *et al.*, 2014) and in vivo (Poznanski *et al.*, 2013).

For each well in which a crystal was observed, the calculated pI was compared with the assigned pH. The protein sequences were considered in groups, as defined in Section 2.2, reflecting the maximum stage in the structure determination pipeline that was attained. The protein structure was determined and deposited in the PDB for the 61 protein sequences in group 1. However, in addition to the conditions that led to the final structure, we also have information about other conditions that produced crystals. Analysis shows that crystals are only obtained in conditions with a pH within one unit of the pI for 9 of the 61 sequences. A total of 28 sequences only result in crystals within two pH units of the pI, 45 sequences only result in crystals within three pH units, 57 sequences only result in crystals within four pH units and the final four proteins crystallize up to five pH units away from the pI. Thus, for over 70% of these protein sequences, crystals are only obtained in experiments buffered within three pH units of the pI.

**Fig. 5.**
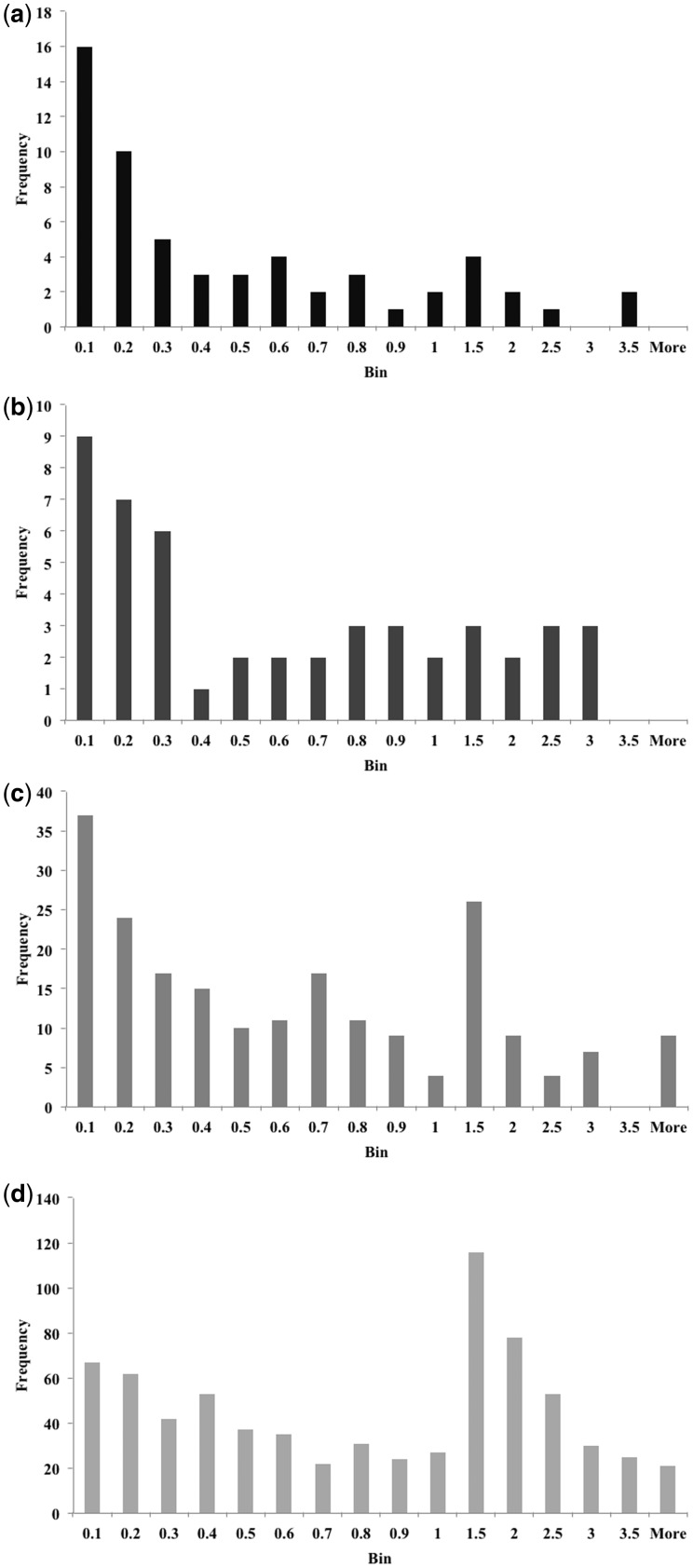
Histograms showing the absolute difference between the pI and the closest pH at which crystals were obtained for proteins in the SGC dataset. (a) shows group 1 (structure determined), (b) shows group 2 (diffraction to at least 3.6 Å), (c) shows group 3 (at least one protein crystal) and (d) shows group 4 (crystal to be followed up)

Particularly in cases when available protein is limited, it is important to identify suitable conditions in as few trials as possible and restricting screening to a particular pH range would reduce the number required. Promising initial conditions (including the pH) could then be optimized to obtain crystals suitable for crystallographic studies (Jancarik and Kim, 1991). For the 61 proteins in the SGC dataset that resulted in a structure deposited in the PDB, we found a correlation of 0.8 between the pH of any crystalline result and the pH at which the final structure was obtained. We therefore investigated the differences between a protein’s isoelectric point and the closest pH value for any conditions producing crystals. Again the proteins were considered in the four groups according to the stage reached in the crystallization pipeline. For those proteins in group 1, 84% crystallized within one pH unit of their pI and 95% crystallized within two pH units of their pI. Crystals were found within one pH unit of their pI for 78% of proteins in group 2 and within two pH units for 88%. In group 3, 74% of proteins crystallized within one pH unit of their pI and 90% within two pH units and for group 4 proteins, 55% produced crystals within one pH unit of their pI and 82% within two pH units. Overall, 85% of proteins did produce crystals within two pH units of their pI. Histograms showing the distribution of shortest distances for each group are given in Figure 5. It is worth noting that those proteins for which no crystals were found within three pH units of their pI (6% of all protein sequences here) tended to have more extreme isoelectric points. Of the 64 such proteins, 46 had a pI outside the range 5–9 and of the 18 protein sequences with a pI in this range, only one with a pI of 7.9 is within the range 6–8.

The 14 proteins in the AZ dataset (Table 1) were used to further test the relationship between pI and the pH of successful crystallization. Once the best crystallization components had been determined for a particular protein, a fine sampling of pH was performed in a 96-well plate with the chosen components buffered between pH 4.5 and pH 9.5. Supplementary Figure S1 shows that crystals were obtained within one pH unit towards neutral from their pI for 11 of the 14 proteins and 13 of 14 crystallize within one pH unit either side of their pI. Only one protein, with a pI of 5.18, did not crystallize within two pH units of its pI.

The stochastic nature of protein crystallization compounds the difficulties of pattern recognition. Supplementary Figure S1 shows that, although several proteins crystallize across a wide range of pH values, crystals are not seen in every 0.25 bin within that range. Reproducibility in screening has been investigated and the results suggest that replication could improve success rates in crystallization experiments (Newman *et al.*, 2007).

## 4 Discussion and conclusion

Linear regression modelling revealed groups of chemicals with similar effects on the pH of a crystallization experiments. The simplest models were obtained for salts with no hydrogen ions and neutral organic compounds. Although a simple linear regression model can be used to relate the pH of the experiment to the buffer pH for both of these chemical groups, the model is different for each group, with the constant offset larger for organics than that for salts. For other groups, the effect of the additional chemical on the buffer pH depends on the concentration of that chemical. In the case of PEGs, the chemical concentration does not appear as a separate variable, but the interaction term between buffer pH and the chemical concentration is significant. It known that PEGs degrade over time (Hampton, 2012; Ray and Puvathingal, 1985; Jurnak, 1986), increasing the acidity of the solution. Similarly, ammonia-containing compounds slowly release the ammonia and affect the pH of a condition (Mikol *et al.*, 1989; Newman *et al.*, 2012). Thus ammonia-containing compounds can become more acidic than PEGs, which when fresh and correctly stored are close to neutral pH, and like the final two groups (acids and basic) require the full linear regression model including the interaction term to represent the pH of the experiment. The last two groups either contain hydrogen ions that have a large impact on pH or contain a hydroxide group, with a large but opposite effect on pH. The largest errors in prediction are due to chemicals that undergo degradation. The deterioration of chemicals, such as PEGs, cannot be predicted but should be considered and storage conditions such as light exposure and temperature could perhaps be controlled.

The grouping of chemicals according to their effect on the pH of a solution means that individual models are not required for each chemical and the effect of chemicals for which there are no examples in the training set can be predicted from the model for the appropriate group. Moreover, the increase in the number of examples available for each model reduces overfitting of the training data and provides more robust models for prediction. Using the chemical grouping suggested by linear regression modelling, the most accurate results were obtained using a single-layer neural network with five nodes. However, the method is less intuitive, and similar results were obtained using the regression equations.

Although we have used our modelling to predict a more accurate pH for the mother liquor, the same method can also be used to predict the pH within the crystallization drop by combing the buffering components. Our results with lysozyme show that the protein itself does not affect the pH unless used in higher concentrations than would typically be used (as shown in Supplementary Table S4).

The ability to predict the effect of different combinations of chemicals on the pH of an experiment allows information in databases such as the PDB to be used in data-mining studies that aim to reduce the number of crystallization trials required. Over the last decade, a number of investigations have considered a possible link between the pI of a protein and the pH at which it will crystallize (Charles *et al.*, 2006). However, such a link has also been disputed (Huber and Kobe, 2004) and it has also been suggested that ‘the pI value of a protein should be avoided when choosing the pH for a protein solution’ (Zhang *et al.*, 2013). Zhang *et al.* (2013) also discuss the issue of the recorded pH not necessarily being the pH of the experimental conditions. Previous findings have been based on the pH of the buffer solution, which can differ from the actual pH by more than three pH units (Bukrinsky and Poulsen, 2001; Newman *et al.*, 2012). Using more accurate pH values that take into account how the concentrations of the various chemicals in the crystallization cocktail affect the pH of the buffer solution, we have shown that there is a relationship between a protein’s pI and the pH under which it will crystallize. In addition to data for the conditions leading to protein structure solution, we have considered the pH of experiments producing crystals that may not have been confirmed as diffraction quality. We found that proteins frequently crystallize within one pH unit of their pI and that 85% of the proteins produced crystals within two pH units of their pI. In most cases, proteins tended to crystallize at a more neutral pH with acidic proteins crystallizing above their pI and basic proteins below their pI, confirming those results found previously (Kantardjieff *et al.*, 2004; Charles *et al.*, 2006). As the majority of proteins are of an acidic pI (Berman *et al.*, 2000), we therefore suggest that a useful initial pH for crystallization trials can be obtained from the pI of the protein in question, but this pH should not simply be taken as that of the buffer solution but, if not measured, should be adjusted to take into account the effect of any additional chemicals. As some proteins do not crystallize close to their pI, we investigated protein properties to determine whether or not such proteins could be predicted. In addition to pI, the GRAVY and the number of D, C, G, H, M, F, P, S, T, W, Y residues (Overton *et al.*, 2008) were calculated for each sequence in the SGC dataset. These properties were used in unsupervised analyses, including k-means clustering and multidimensional scaling, but we were unable to determine any association between the sequence properties and the proteins propensity to crystallize at a pH close to its pI. Differences in physical crystallization conditions such as temperature and whether the protein sample was frozen were not taken into account, although analysis showed there were sequences that resulted in protein structures at 4 and room temperature, for both fresh and frozen protein samples with and without ligands.

It is interesting to note that two proteins in the AZ dataset, Glycolytic enzyme A and Glycolytic enzyme D, have only the single surface amino acid change E193A and E193D, respectively, but have very different crystallization patterns. Although Glycolytic enzyme D crystallizes within a limited pH range, Glycolytic enzyme A crystallizes across a wide range of pH values. This supports the view of Dale *et al.* (2003) that the protein itself is an important variable in crystallization and rational mutagenesis may aid the screening process.

## Supplementary Material

Supplementary Data
