# Supplementary material for: Using isoelectric point to determine the pH for initial protein crystallization trials
Source: Bioinformatics. 2015 Jan 7;31(9):1444–51. doi: 10.1093/bioinformatics/btv011 (PMC4410668; doi:10.1093/bioinformatics/btv011)
Supplement: Supplementary Data [file supp_31_9_1444__index.html]

Using isoelectric point to determine the pH for initial protein crystallization trials — Using isoelectric point to determine the pH for initial protein crystallization trials — Supplementary Data 

# Using isoelectric point to determine the pH for initial protein crystallization trials

## Supplementary Data

files

**Files in this Data Supplement:**

- Supplementary Data - xlsx file
- Supplementary Data - docx file
- Supplementary Data - docx file
- Supplementary Data - docx file
- Supplementary Data - docx file
- Supplementary Data - docx file
- Supplementary Data - docx file
- Supplementary Data - docx file
