# Supplementary material for: Using isoelectric point to determine the pH for initial protein crystallization trials
Source: Bioinformatics. 2015 Jan 7;31(9):1444–51. doi: 10.1093/bioinformatics/btv011 (PMC4410668; doi:10.1093/bioinformatics/btv011)
Supplement: Supplementary Data [file supp_btv011_Supplementary_Figure_1.docx]

|  |  |  |  |  |  |  |  |  |  |  | **Bin** |  |  |  |  |  |  |  |  |  |  |
| --- | --- | --- | --- | --- | --- | --- | --- | --- | --- | --- | --- | --- | --- | --- | --- | --- | --- | --- | --- | --- | --- |
|  | **pI** | **4.75** | **5** | **5.25** | **5.5** | **5.75** | **6** | **6.25** | **6.5** | **6.75** | **7** | **7.25** | **7.5** | **7.75** | **8** | **8.25** | **8.5** | **8.75** | **9** | **9.25** | **9.5** |
| Glycolytic A | **7.52** |  |  |  |  | ◊ | ◊ | ◊ | ◊ | ♦ | ♦ | ♦ | ♦ | ◊ | ◊ |  |  |  |  |  |  |
| Glycolytic D | **6.75** |  |  |  |  |  | ♦ |  | ♦ |  |  |  |  |  |  |  |  |  |  |  |  |
| Glycolytic wt | **6.75** |  |  |  |  | **♦** |  |  |  |  |  |  |  |  |  |  |  |  |  |  |  |
| α- Chymo A | **8.52** |  |  |  |  |  | ◊ | ◊ | ◊ | ◊ | ◊ | ◊ | ◊ | ♦ | ♦ | ♦ |  |  |  |  |  |
| Bovine Catalase | **6.79** |  |  |  |  |  |  | ◊ | ◊ | ◊ | ♦ | ♦ | ♦ | ♦ |  | ♦ | ♦ | ♦ |  | ◊ |  |
| Protease K212A | **4.93** |  |  |  |  |  | ♦ | ◊ | ◊ | ◊ | ◊ | ◊ | ◊ | ◊ |  |  |  |  |  |  |  |
| Protease K234A | **5.03** |  |  | ♦ | ♦ | ♦ | ♦ | ◊ | ◊ | ◊ | ◊ | ◊ | ◊ | ◊ |  |  |  |  |  |  |  |
| Protease K249A | **5.03** | ♦ | ♦ | ♦ | ♦ | ♦ | ♦ | ◊ | ◊ | ◊ |  |  |  |  |  |  |  |  |  |  |  |
| Protease E171A | **5** |  |  |  |  |  | ♦ | ◊ | ◊ | ◊ | ◊ | ◊ |  |  |  |  |  |  |  |  |  |
| Concanavalin A | **5.47** |  | ♦ | ♦ | ♦ | ♦ | ♦ | ♦ | ♦ | ◊ | ◊ | ◊ | ◊ | ◊ | ◊ |  |  |  |  |  |  |
| Lysozyme | **9.36** |  |  | ◊ | ◊ | ◊ |  |  |  | ◊ |  |  |  |  |  |  |  |  | ♦ |  |  |
| Kinase 1 | **5.18** |  |  |  |  |  |  |  |  |  | ◊ | ◊ | ◊ | ◊ |  |  |  |  |  |  | ◊ |
| Pig Trypsin | **7** |  |  |  |  |  | ◊ | ♦ | ♦ | ♦ | ♦ | ♦ | ♦ | ♦ | ♦ | ◊ | ◊ | ◊ |  |  |  |
| Thaumatin | **8.46** |  |  |  |  | ◊ | ◊ | ◊ | ◊ | ◊ | ◊ | ◊ | ◊ | ♦ | ♦ | ♦ | ♦ | ♦ | ♦ | ♦ | ♦ |

| ♦ | Crystallised within one unit of its pI towards a neutral pH |
| --- | --- |
| ♦ | Crystallised within one unit of its pI away from a neutral pH |
| ◊ | Crystallised elsewhere |

**Supplementary Figure 1: Distribution of crystals for AZ proteins.** The 0.25 pH bin in which each of the fourteen AstraZeneca proteins crystallised is indicated with a diamond. Crystals obtained within one unit of the pI towards a neutral pH are indicated in green. Similarly, crystals within one unit of the pI but away from a neutral pH are shown in red. Of the 14 proteins, 11 did crystallise within one unit of the pI towards a neutral pH, a further two glycolytic enzymes, D and wt, crystallised within one unit of their pI but away from a neutral pH. Only Kinase 1 crystallised more than one unit away from its pI.
