# Supplementary material for: Using isoelectric point to determine the pH for initial protein crystallization trials
Source: Bioinformatics. 2015 Jan 7;31(9):1444–51. doi: 10.1093/bioinformatics/btv011 (PMC4410668; doi:10.1093/bioinformatics/btv011)
Supplement: Supplementary Data [file supp_btv011_Supplementary_Figure_2.docx]

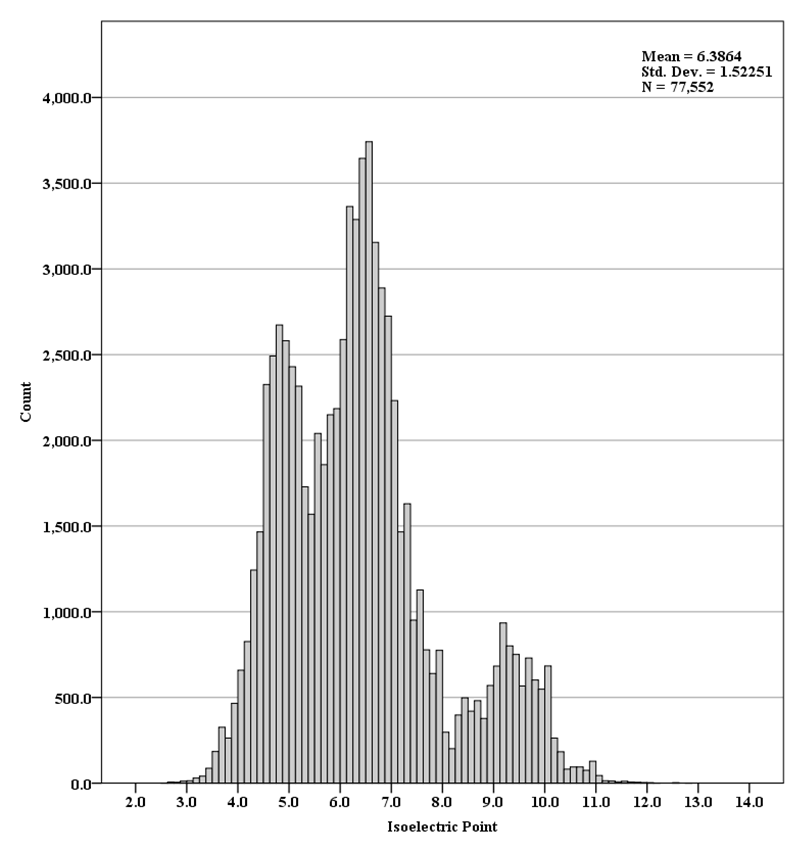
**Supplementary Figure 2.** Histogram showing pI values for 77,552 proteins in the PDB. The values are calculated from sequences using PROPKA. In contrast to the trimodal distribution seen here, analysis of 15906 conditions from 256 commercial screens suggests that buffer pH has a normal distribution with mean value pH 6.5.
