# Supplementary material for: Using isoelectric point to determine the pH for initial protein crystallization trials
Source: Bioinformatics. 2015 Jan 7;31(9):1444–51. doi: 10.1093/bioinformatics/btv011 (PMC4410668; doi:10.1093/bioinformatics/btv011)

**Supplementary Information: Linear regression modelling**

For each chemical group (dihydrogen salts, ammonia-containing, hydroxide salts, organic, PEG, salt or salt of weak acid), the full model was initially

$\hat{\boldsymbol{pH}_{\boldsymbol{S}}}\boldsymbol{=}\boldsymbol{\beta}_{\boldsymbol{0}}\boldsymbol{+}\boldsymbol{\beta}_{\boldsymbol{1}}\boldsymbol{B+}\boldsymbol{\beta}_{\boldsymbol{2}}\boldsymbol{log}_{\boldsymbol{10}}\boldsymbol{C+}\boldsymbol{\beta}_{\boldsymbol{3}}\boldsymbol{B\cdot}\boldsymbol{log}_{\boldsymbol{10}}\boldsymbol{C}$.

Stepwise variable selection using Bayesian Information Criterion (BIC) was performed in the R programming environment using the commands

fullmodel = lm(x[,1] ~ x[,2] + x[,3] + x[,4])

n= nrow(x)

step(fullmodel, k = log(n))

where x[,1] is the pH (the response, $\hat{\mathrm{pH}_{S}}$ , x[,2] is buffer concentration (B), x[,3] is the logarithm (base 10) of the chemical concentration ($\log_{10}C)$and x[,4] is the interaction term between these two covariates. The use of k = log(n) ensures that BIC is used rather than Akaike Information Criterion (AIC) although it is still labelled AIC in the output.

Simplified models were obtained for some chemical groups as shown in the R output below.

**Dihydrogen salts**

model:

$$\hat{{pH}_{S}}=1.74+{0.80}_{1}B+0.71{log}_{10}C-0.21B\cdot{log}_{10}C$$

R output:

**
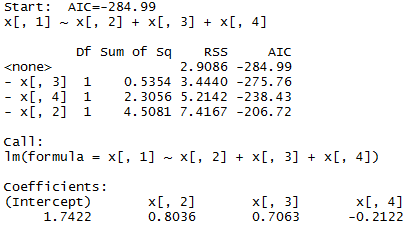
**

**Ammonia**

model:

$$\hat{{pH}_{S}}=0.74+{0.92}_{1}B+1.06{log}_{10}C-0.16B\cdot{log}_{10}C$$

R output:


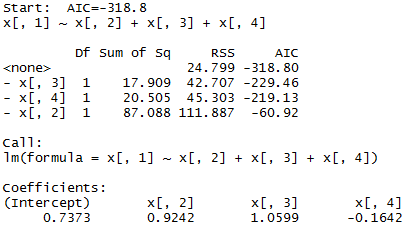


**Hydroxide salts**

model:

$$\hat{{pH}_{S}}=-6.55+{1.83}_{1}B+4.03{log}_{10}C-0.48B\cdot{log}_{10}C$$

R output:


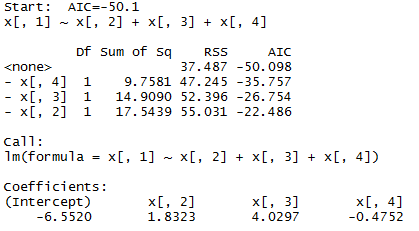


**organic**

model:

$$\hat{{pH}_{S}}=1.67+{0.71}_{1}B$$

R output:

**
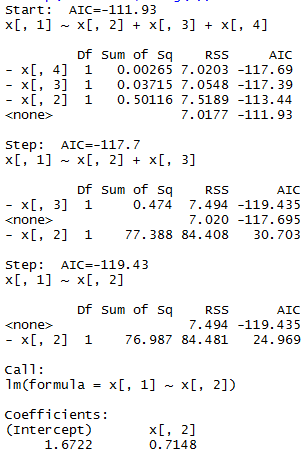
**

**PEGs**

model:

$$\hat{{pH}_{S}}=1.91+{0.72}_{1}B-0.03B\cdot{log}_{10}C$$

R output:

**
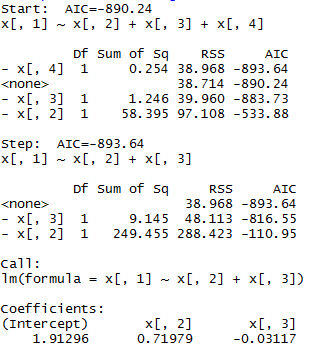
**

**salts**

model:

$$\hat{{pH}_{S}}=1.18+0.87B$$

R output:

**
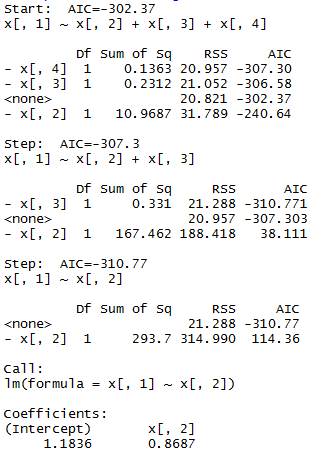
**

**acid salt**

model:

$$\hat{{pH}_{S}}=0.204+{1.01}_{1}B+0.998{log}_{10}C-0.129B\cdot{log}_{10}C$$

R output:


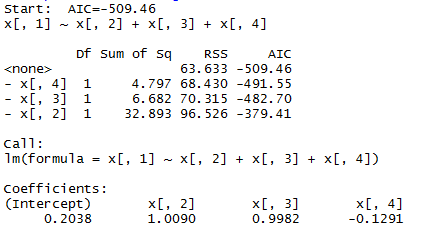

Supplement: Supplementary Data [file supp_btv011_Supplementary_Information.docx]
