# Supplementary material for: Using isoelectric point to determine the pH for initial protein crystallization trials
Source: Bioinformatics. 2015 Jan 7;31(9):1444–51. doi: 10.1093/bioinformatics/btv011 (PMC4410668; doi:10.1093/bioinformatics/btv011)
Supplement: Supplementary Data [file supp_btv011_Supplementary_Table_1.docx]

|  | **dihydrogen salts** | **ammonia** | **hydroxide salts** | **organic** | **peg** | **salt** | **salt of weak acid** |
| --- | --- | --- | --- | --- | --- | --- | --- |
|  | ammonium dihydrogen phosphate | ammonium acetate | potassium phosphate dibasic | 1,2-propanediol | jeffamine ed-2001 | cadmium chloride | calcium acetate |
|  | potassium dihydrogen phosphate | ammonium citrate tribasic | sodium citrate tribasic | 1,4-butanediol | jeffamine ed-2003 | caesium chloride | dl- malic acid |
|  | sodium dihydrogen phosphate | ammonium phosphate dibasic | sodium phosphate dibasic | 2,3-butanediol | jeffamine m-600 | calcium chloride | magnesium acetate |
|  |  | ammonium |  | 2-propanol | pentaerythritol ethoxylate (15/4 eo/oh) | cobalt chloride | magnesium formate |
|  |  | ammonium tartrate dibasic |  | dioxane | pentaerythritol propoxylate (5/4 po/oh) | lithium chloride | magnesium |
|  |  |  |  | dmso | polypropylene glycol p 400 | lithium sulphate | malonate |
|  |  |  |  | ethanol | polyvinylpyrrolidone k 15 | l-proline | potassium sodium tartrate |
|  |  |  |  | ethylene glycol | tmsulphone | magnesium chloride | potassium sodium tartrate |
|  |  |  |  | glycerol | pegs of various molecular weights | magnesium | sodium acetate |
|  |  |  |  | hexanediol | monomethyl ether pegs of various molecular weights | magnesium chloride | sodium formate |
|  |  |  |  | mpd | dimethyl ether pegs of various molecular weights | manganese chloride | sodium malonate |
|  |  |  |  | reagent alcohol |  | nickel chloride | succinic acid |
|  |  |  |  |  |  | polyacrylic acid 5100 | tacsimate |
|  |  |  |  |  |  | potassium chloride | zinc acetate |
|  |  |  |  |  |  | sodium chloride |  |
|  |  |  |  |  |  | sodium sulphate |  |
|  |  |  |  |  |  | trimethylamine n-oxide |  |
| β_0_ | 1.74 | 0.74 | -6.55 | 1.67 | 1.91 | 1.18 | 0.20 |
| β_1_ | 0.80 | 0.92 | 1.83 | 0.71 | 0.72 | 0.87 | 1.01 |
| β_2_ | 0.71 | 1.06 | 4.03 | 0.00 | 0.00 | 0.00 | 1.00 |
| β_3_ | -0.21 | -0.16 | -0.48 | 0.00 | -0.03 | 0.00 | -0.13 |

**Supplementary Table 1: Grouping of the chemical species from the 5161 conditions** The seven groups identified by similarity of regression models with the regression coefficients shown at the bottom of the table.
