# Supplementary material for: Using isoelectric point to determine the pH for initial protein crystallization trials
Source: Bioinformatics. 2015 Jan 7;31(9):1444–51. doi: 10.1093/bioinformatics/btv011 (PMC4410668; doi:10.1093/bioinformatics/btv011)
Supplement: Supplementary Data [file supp_btv011_Supplementary_Table_2.docx]

| **dihydrogen salts** | **ammonia** | **hydroxide salts** | **organic** | **peg** | **salt** | **salt of weak acid** |
| --- | --- | --- | --- | --- | --- | --- |
| sodium phosphate monobasic | ammonium acetate | potassium phosphate dibasic | 1,2-propanediol | jeffamine ed-2001 | cadmium chloride | calcium acetate |
|  | ammonium chloride |  | 2‐propanol | jeffamine m-600 | caesium chloride | lithium sulphate |
|  | ammonium citrate |  | dioxane | peg 1000 | calcium chloride | magnesium formate |
|  | ammonium phosphate |  | ethylene glycol | peg 10000 | cobalt chloride | magnesium sulphate |
|  | ammonium sulphate |  | glycerol | peg 2000 mme | lithium chloride | potassium citrate |
|  |  |  | mpd | peg 300 | magnesium chloride | sodium formate |
|  |  |  | tmao | peg 3350 | nickel chloride | sodium malate |
|  |  |  |  | peg 6000 | potassium thiocyanate | sodium malonate |
|  |  |  |  | polyvinylpyrrolidone 40 k | sodium bromide | sodium succinate |
|  |  |  |  |  | sodium chloride | zinc acetate |
|  |  |  |  |  | sodium nitrate |  |

**Supplementary Table 2: Grouping of the chemical species from the JCSG +4 conditions.** The chemicals found in the screen are assigned to one of the predictive groups.
