# Supplementary material for: Using isoelectric point to determine the pH for initial protein crystallization trials
Source: Bioinformatics. 2015 Jan 7;31(9):1444–51. doi: 10.1093/bioinformatics/btv011 (PMC4410668; doi:10.1093/bioinformatics/btv011)
Supplement: Supplementary Data [file supp_btv011_Supplementary_Table_3.docx]

|  | Solution 1 | Solution 2 | Solution 3 |
| --- | --- | --- | --- |
| ratio protein:buffer | (40mg/ml lysozyme | (40mg/ml lysozyme | 40mg/ml lysozyme |
|  | 100mM NaCl | 100mM NaCl | 100mM NaCl |
| added to… | 50mM PCTP at pH 5 | 50mM PCTP at pH 7 | 50mM PCTP at pH 9 |
| 50mM PCTP pH 5 in ratio 1:1 |  | 6.26 | 7.06 |
|  |  | 6.25 | 7.05 |
|  |  | 6.24 | 7.06 |
| 50mM PCTP pH 5 in ratio 2:1 |  | 6.5 | 7.62 |
|  |  | 6.53 | 7.62 |
|  |  | 6.53 | 7.61 |
| 50mM PCTP pH 7 in ratio 1:1 | 5.84 |  | 7.97 |
|  | 5.82 |  | 7.98 |
|  | 5.85 |  | 7.98 |
| 50mM PCTP pH 7 in ratio 2:1 | 5.42 |  | 8.37 |
|  | 5.48 |  | 8.37 |
|  | 5.49 |  | 8.38 |
| 50mM PCTP pH 9 in ratio 1:1 | 6.46 | 7.82 |  |
|  | 6.44 | 7.8 |  |
|  | 6.44 | 7.8 |  |
| 50mM PCTP pH 9 in ratio 2:1 | 5.84 | 7.46 |  |
|  | 5.88 | 7.47 |  |
|  | 5.88 | 7.46 |  |

**Supplementary Table 3: pH within the Crystallisation Drop.** The table shows the measured pH of the components of the crystallisation drop for varying ratios of protein solution: mother liquor.
