# Supplementary material for: Using isoelectric point to determine the pH for initial protein crystallization trials
Source: Bioinformatics. 2015 Jan 7;31(9):1444–51. doi: 10.1093/bioinformatics/btv011 (PMC4410668; doi:10.1093/bioinformatics/btv011)
Supplement: Supplementary Data [file supp_btv011_Supplementary_Table_4.docx]

| **10mM Buffer-vs-Crystallant** | | | | | | |
| --- | --- | --- | --- | --- | --- | --- |
|  | **pH5-vs-pH7** | **pH5-vs-pH9** | **pH7-vs-pH5** | **pH7-vs-pH9** | **pH9-vs-pH5** | **pH9-vs-pH7** |
| 3:1 | 6.53 | 7.86 | 5.36 | 8.5 | 5.66 | 7.07 |
| 2:1 | 6.67 | 8.15 | 5.31 | 8.64 | 5.48 | 6.99 |
| 1:1 | 6.69 | 8.51 | 5.17 | 8.78 | 5.17 | 6.89 |
| 1:2 | 6.79 | 8.72 | 5.05 | 8.85 | 5 | 6.92 |
| 1:3 | 6.82 | 8.79 | 5.02 | 8.86 | 4.99 | 6.92 |
| **10mM Buffer + 40mgml Lysozyme-vs-Crystallant** | | | | | | |
|  | pH5-vs-pH7 | pH5-vs-pH9 | pH7-vs-pH5 | pH7-vs-pH9 | pH9-vs-pH5 | pH9-vs-pH7 |
| 3:1 | 6.37 | 7.37 | 5.48 | 8.11 | 5.74 | 7.09 |
| 2:1 | 6.42 | 7.8 | 5.39 | 8.44 | 5.61 | 7.1 |
| 1:1 | 6.63 | 8.21 | 5.22 | 8.66 | 5.33 | 6.98 |
| 1:2 | 6.72 | 8.57 | 5.08 | 8.77 | 5.17 | 6.97 |
| 1:3 | 6.8 | 8.66 | 5.07 | 8.79 | 5.11 | 6.95 |
| **Absolute Difference with and without Lysozyme** | | | | | | |
| delta for protein | pH5-vs-pH7 | pH5-vs-pH9 | pH7-vs-pH5 | pH7-vs-pH9 | pH9-vs-pH5 | pH9-vs-pH7 |
| 3:1 | 0.16 | 0.49 | 0.12 | 0.39 | 0.08 | 0.02 |
| 2:1 | 0.25 | 0.35 | 0.08 | 0.2 | 0.13 | 0.11 |
| 1:1 | 0.06 | 0.3 | 0.05 | 0.12 | 0.16 | 0.09 |
| 1:2 | 0.07 | 0.15 | 0.03 | 0.08 | 0.17 | 0.05 |
| 1:3 | 0.02 | 0.13 | 0.05 | 0.07 | 0.12 | 0.03 |

**Supplementary Table 4: pH within the Crystallisation Drop.** The table shows the measured pH of the components of the crystallisation drop for varying ratios of protein buffer solution: mother liquor, with and without lysozyme.
